# An unsupervised cluster analysis of multimorbidity patterns in older adults in Shenzhen, China

**DOI:** 10.3389/fpubh.2025.1557721

**Published:** 2025-06-06

**Authors:** Xiaolong Guo, Peiyi Liu, Jing Guo, Naiwen Zhang, Haiyan Huang, Jianjun Liu, Zhen Tan, Guo Dan

**Affiliations:** ^1^School of Biomedical Engineering, Shenzhen University Medical School, Shenzhen University, Shenzhen, China; ^2^Shenzhen Key Laboratory of Modern Toxicology, Shenzhen Medical Key Discipline of Health Toxicology, Shenzhen Center for Disease Control and Prevention, Shenzhen, China; ^3^Endocrinology and Metabolism, Shenzhen University General Hospital, Shenzhen, China; ^4^Health Management Center, Shenzhen University General Hospital, Shenzhen University, Shenzhen, China

**Keywords:** multimorbidity, multimorbidity pattern, unsupervised learning, older adults, Shenzhen city

## Abstract

**Background:**

Population aging challenges health care systems due to the high prevalence and impact of multimorbidity in older adults. Studies on multimorbidity in Shenzhen have primarily focused on the quantity of multimorbidity, lacking in-depth exploration of multimorbidity patterns.

**Methods:**

Based on baseline data from the Shenzhen aging-related disease cohort, this study analyzed information from 8,911 people aged 60 and above after excluding missing and abnormal values from interview results. Using self-organizing map combined with weighted k-means, the distribution of diseases in the population was visualized, dividing the overall population into four clusters. The study also analyzed comorbidity and association rules for each cluster.

**Result:**

This study found a high prevalence of cardiometabolic comorbidities among the older adult in Shenzhen, reaching 15.83%, and detailed the distribution of specific comorbidity combinations. Hypertension had a high prevalence and was the most common factor in comorbidities among Shenzhen’s older adult. Additionally, hyperuricemia was included as a disease to explore its multimorbidity patterns with other chronic conditions.

**Conclusion:**

The study found that multimorbidity is prevalent among the older adult in Shenzhen and explored their patterns, suggesting that Shenzhen should enhance screening and integrated management of high-risk groups and implement public health interventions to alleviate the multimorbidity burden.

## 1 Introduction

With increased life expectancy and declining fertility rates, the global population is rapidly aging (1). Concurrently, as populations age and lifestyles change, multimorbidity has become increasingly prevalent. Multimorbidity, typically referring to the coexistence of two or more chronic diseases in an individual, has garnered significant attention in recent years. Globally, approximately one-third of adults including a substantial proportion in low- and middle-income countries and more than half of all adults with any chronic disease suffer from multimorbidity (2). Among the global adult population aged 60 and above, over half are afflicted with multimorbidity (51.0, 95% CI: 44.1–58.0%) (3).

Multimorbidity is associated with numerous adverse health outcomes, such as increased mortality, disability, reduced quality of life, and elevated hospitalization rates, thereby posing significant public health challenges globally. In high-income countries, multimorbidity accounts for 78% of primary care consultations (4), and individuals with multimorbidity are hospitalized more frequently and for longer durations than those with one or no diseases (5). Moreover, there is an almost exponential relationship between the number of chronic diseases and the associated healthcare costs. Notably, the COVID-19 pandemic has also exposed vulnerabilities in global public health systems (6–10). During the pandemic, individuals with multimorbidity were at higher risk of infection and adverse outcomes, including hospitalization (10, 11).

In China, the management of chronic diseases, particularly multimorbidity, has received considerable attention from the government and health departments. Several studies have explored multimorbidity, including large-scale research such as analyses among two million Chinese adults and studies on the relationship between multimorbidity patterns and mortality involving 500,000 Chinese adults (12, 13). A recent systematic review indicated that nearly one-quarter of Chinese adults suffer from multimorbidity, with prevalence increasing rapidly with age (14). However, most studies on multimorbidity in China are based on medical data from a single city or province (14–18). Due to differences in economic levels, healthcare services, and population health quality among different regions in China, variations in the prevalence and patterns of diseases and multimorbidity exist, necessitating more specific analyses.

Shenzhen is one of the fastest-growing cities in China and is widely regarded as the “City of Innovation,” with technology, finance, and manufacturing as its pillar industries. It has transformed from a fishing village into a modern metropolis within just a few decades. As one of China’s first Special Economic Zones, Shenzhen has attracted talent from across the country and around the world, resulting in a highly diverse population. Although youthfulness has long been considered a defining characteristic of the city, projections suggest that Shenzhen will officially enter an aging society by 2029. As a representative southern Chinese city, several studies on multimorbidity have been conducted in Shenzhen to date (19–22). A large-scale cross-sectional study published in 2023 investigated the prevalence of multimorbidity among the older adult population in Shenzhen. The study demonstrated that the prevalence rates of obesity, hypertension, diabetes, anemia, chronic kidney disease, hyperuricemia, dyslipidemia, and fatty liver disease were 10.41, 62.09, 24.21, 12.78, 6.14, 20.52, 44.32, and 33.25%, respectively. The prevalence of multimorbidity was 63%, with an average of 2.14 chronic diseases per participant (22). The study also analyzed predictors of multiple diseases. However, most studies on multimorbidity in Shenzhen have primarily focused on the quantity of multimorbidity, lacking in-depth exploration of multimorbidity patterns.

Machine learning, particularly unsupervised clustering techniques, has been utilized to investigate patterns of multimorbidity within specific populations. Various methods have been employed, including Principal Component Analysis (PCA), Apriori algorithms, hierarchical clustering, and k-means clustering (23–26). In recent years, Self-Organizing Maps (SOM), a visualization-oriented unsupervised learning method, have also been applied to multimorbidity analysis, often in conjunction with hierarchical clustering and k-means clustering (27–29). This study proposes a novel unsupervised learning approach that combines SOM with Weighted k-means (W-k-means) to identify multimorbidity patterns among the older adult in Shenzhen. The primary objective is to identify disease groupings or clusters within Shenzhen’s older adult population and to further characterize and describe patient profiles observed both in the overall population and within these clusters. This involves exploring common comorbidity combinations and the most common causes of multimorbidity among the older adult, aiming to provide valuable information for health policy formulation and the allocation of health services.

## 2 Methods

The data for this cross-sectional study were derived from the baseline data of the Shenzhen aging-related disease cohort established between 2017 and 2018 (30). This dataset includes 9,411 older adult participants aged 60 to 92 years from 51 community health service centers in Luohu District, Shenzhen. The inclusion and exclusion criteria have been previously published (30). All participants agreed to join in the cohort and provide informed written consent. The study has been approved by the Review Board of Shenzhen Center for Disease Control and Prevention (approval numbers: R2017001 and R2018020).

Data collected included demographic and socioeconomic information, lifestyle factors, medical history, family history of major non-communicable chronic diseases, environmental exposures, clinical analyses of blood and urine, imaging measurements, anthropometric measurements, and neurological and mental health assessments. Demographic data (e.g., name, ID number, gender, date of birth, education level) and chronic disease history (including hypertension, dyslipidemia, coronary heart disease, stroke, diabetes, cancer, neurological and psychiatric diseases) were collected using semi-structured questionnaires. The age range was calculated based on birth year reported in 5-year intervals, and the data was divided into four groups. Older adult individuals aged over 76 were classified as older seniors, and since the data volume was not suitable for division into 5-year intervals, they were grouped together. Body mass index (BMI) was categorized as underweight (BMI < 18.5), normal weight (BMI ≥ 18.5 and <25), overweight (BMI ≥ 25 and <30), and obese (BMI ≥ 30).

In this study, multimorbidity was defined as the simultaneous presence of two or more chronic diseases. A total of 21 chronic diseases were used to assess multimorbidity, including hyperuricemia, chronic bronchitis, chronic obstructive pulmonary disease (COPD), asthma, pulmonary tuberculosis, hypertension, hyperlipidemia, angina pectoris, myocardial infarction, coronary heart disease, chronic hepatitis, nephritis, diabetes, migraine, stroke, Alzheimer’s disease, Parkinson’s disease, depression, osteoporosis, arthritis, and tumors. These 21 diseases encompass the majority of non-communicable chronic conditions associated with aging, including neurological disorders, mental illnesses, chronic respiratory diseases, cardiovascular diseases, metabolic disorders, cancers, injuries, and other non-communicable diseases. They can be used to describe the patterns of morbidity and multimorbidity among the older adult population in Shenzhen. Based on blood analysis results, males with uric acid levels exceeding 420 μmol/L and females exceeding 360 μmol/L were considered to have hyperuricemia (31–34). Each disease is derived from the patient’s medical history report, based on which a wide-format high-dimensional binary matrix is created for unsupervised clustering analysis. Each column corresponds to a diagnostic category, with “1” indicating the presence of the condition and “0” indicating its absence. Based on these disease characteristics and demographic features, patients with multiple missing values and subjects with incorrectly coded features were excluded, resulting in a final sample of 8,911 subjects.

Cardiometabolic multimorbidity refers to the coexistence of two or more cardiometabolic diseases, including hypertension, diabetes, coronary heart disease, stroke, and dyslipidemia. Additionally, multimorbidity focuses on the overall coexistence of multiple diseases, while comorbidity refers to more specific combinations of diseases, emphasizing the relationships and interactions between them.

The primary goal of this research was to identify disease groupings or clusters among older adult patients in Shenzhen and to characterize patient profiles within these clusters. Previous studies on multimorbidity have often employed hierarchical clustering and k-means clustering methods. In this study, we adopted a novel clustering approach to better delineate disease groups. We described the prevalence rates in the overall older adult population and within clusters, identified common comorbidity combinations and performed association rule mining. Social factors and demographic characteristics across clusters were also analyzed. Machine learning and statistical analyses were conducted using R statistical software (v. 4.4.1).

**Figure 1:**
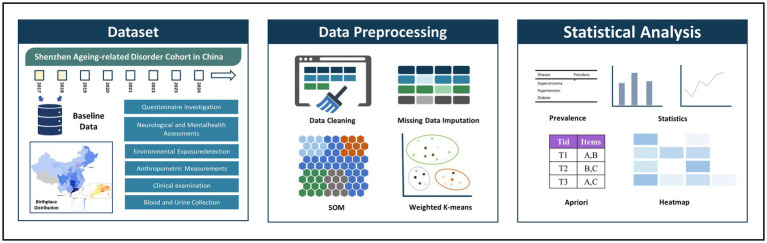
Overview of study approach (30). Map reproduced with permission from “The birthplace distribution of the studied individuals”. By Liu L et al., licensed under CC BY-NC 4.0.

As shown in Figure 1, this study employs a two-stage self-organizing clustering method to determine whether multimorbidity conditions group together. The first stage provides the topological coordinates of prototypes, allowing for clustering in the second stage using classical methods. The Self-Organizing Map (SOM), proposed by Kohonen, is an unsupervised artificial neural network that transforms and visualizes high-dimensional input data onto a low-dimensional map (27, 35–37). SOM preserves the topological structure of the original data, forming a two-dimensional space composed of nodes that cluster relevant data. Input data are connected to a selected lattice of nodes, distributing the dataset across these nodes. The SOM process begins by initializing the grid with random samples from the dataset, then iteratively compares node distances and assigns individuals to nodes.

SOM + K-means is a commonly used method for exploring patterns of multimorbidity. Considering that the traditional K-means algorithm used in the SOM + K-means approach cannot effectively adjust for the distribution of sample sizes across SOM nodes, this study replaces K-means with the Weighted K-means algorithm. In addition to W-K-means, we conducted comparative analyses using SOM + K-means, conventional K-means, Latent Class Analysis (LCA), and Multiple Correspondence Analysis combined with K-means (MCA + K-means). As this study is based on real-world data, there are no ground-truth labels for patient clusters to directly assess clustering performance. Nevertheless, several internal metrics can provide guidance and reference during the clustering process, although the final evaluation ultimately depends on clinical interpretability and practical relevance. Through comparison, the W-K-means approach demonstrated several advantages, including: clinically interpretable clusters; more stable and distinct multimorbidity patterns; a clear gradient in disease burden; and moderate, clinically meaningful cluster sizes. Therefore, we report the clustering results based on the W-K-means method. Regarding the selection of SOM network size N, we chose a network that is large enough to differentiate the data evenly without causing over-dispersion, and adopted a sufficient learning rate to ensure adequate iteration and convergence.

**Figure 2:**
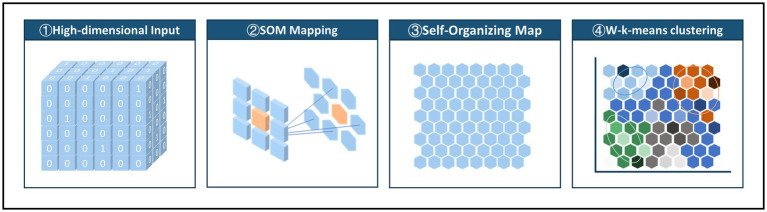
Two-level SOM clustering approach: mapping high-dimensional input data to a low-dimensional SOM network and using node activation frequencies as weights in a weighted K-means clustering.

As shown in Figure 2, in the two-stage clustering method, the first stage involves constructing the SOM network, and the second stage applies W-k-means clustering. The weighted k-means algorithm considers the importance of different data points by assigning weights during clustering. In this study (38), the activation frequency of each node in the SOM network was used as the weight. A 30 × 30 node network with hexagonal topology was initialized to simulate positional relationships within the network. The SOM was trained using an initial learning rate of 0.05 and a final learning rate of 0.01 over 20,000 iterations to ensure stability and convergence. To determine the appropriate number of clusters, we evaluated within-cluster distances for different values and visualized the within sum of squares (WSS). Based on the WSS plot (Supplementary Figure 1), four clusters were selected as the optimal number.

The Apriori algorithm was employed to analyze common combinations of comorbidities among older adult individuals with multimorbidity in both the overall population and the four clusters. The algorithm extracts frequent itemsets from large datasets and generates association rules describing relationships among these items. Key metrics for association rules include support, confidence, and lift. In multimorbidity research, support represents the frequency of co-occurrence of diseases, confidence indicates the probability of one disease occurring given another, and lift measures the strength of the association beyond chance. In this study, the minimum support was set at 3.0%, the minimum confidence at 30%, and the maximum number of antecedent items at three, the parameter settings were adapted from another study on Multimorbidity among older adults in China.

## 3 Results

### 3.1 Overall population

Table 1 presents the disease characteristics among participants with different characteristics. The average age of all study participants was 67.72 ± 5.42 years, with 42.74% being male. A total of 7,373 individuals (82.74%) had an educational level of junior high school or above. There were 1,816 smokers (20.37%) and 1,286 alcohol drinkers (14.43%). Additionally, 47.14% of the participants were overweight or obese. There were 3,220 cases (36.14%) with only one disease, and 3,113 cases (34.93%) had multimorbidity.

**Table 1:** Multimorbidity prevalence in different features.

Features	Patients (*n*, %)	No disease (*n*, %)	Single disease (*n*, %)	Multimorbidity (*n*, %)
Woman	3,809 (42.74%)	1,090 (42.28%)	1,405 (43.63%)	1,314 (42.21%)
Man	5,102 (57.26%)	1,488 (57.72%)	1815 (56.37%)	1799 (57.79%)
Age				
60–65	2,987 (33.52%)	1,028 (39.88%)	1,122 (34.84%)	837 (26.89%)
66–70	3,086 (34.63%)	904 (35.07%)	1,102 (34.22%)	1,080 (34.69%)
71–75	1732 (19.44%)	423 (16.41%)	622 (19.32%)	687 (22.07%)
76-	1,106 (12.41%)	223 (8.65%)	374 (11.61%)	509 (16.35%)
BMI				
BMI < 18.5	208 (2.33%)	107 (4.15%)	75 (2.33%)	26 (0.84%)
18.5 ≤ BMI < 25	4,502 (50.52%)	1,584 (61.44%)	1,621 (50.34%)	1,297 (41.66%)
25 ≤ BMI < 30	3,668 (41.16%)	817 (31.69%)	1,344 (41.74%)	1,507 (48.41%)
BMI ≥ 30	533 (5.98%)	70 (2.72%)	180 (5.59%)	283 (9.09%)
Education				
Primary school and below	1,538 (17.26%)	417 (16.18%)	547 (16.99%)	574 (18.44%)
Junior high school	2,448 (27.47%)	710 (27.54%)	872 (27.08%)	866 (27.82%)
Senior high school	3,027 (33.97%)	891 (34.56%)	1,131 (35.12%)	1,005 (32.28%)
College or undergraduate	1875 (21.04%)	552 (21.41%)	664 (20.62%)	659 (21.17%)
Graduate and above	23 (0.26%)	8 (0.31%)	6 (0.19%)	9 (0.29%)
Smoke				
No	7,095 (79.62%)	2075 (80.49%)	2,556 (79.38%)	2,464 (79.15%)
Yes	1816 (20.38%)	503 (19.51%)	664 (20.62%)	649 (20.85%)
Drink				
No	7,625 (85.57%)	2,209 (85.69%)	2,747 (85.31%)	2,669 (85.74%)
Yes	1,286 (14.43%)	369 (14.31%)	473 (14.69%)	444 (14.26%)

We calculated the average number of diseases corresponding to different ages and BMI categories, and linear trend tests were performed, revealing a significant upward trend in the number of diseases with increasing age and BMI (*p* < 0.0001) (Supplementary Figure 2). Supplementary Figure 3 shows the prevalence of each disease across different characteristics.

As shown in Table 2, among the 21 diseases involved in the study, the top four diseases with prevalence rates greater than 10% are hyperuricemia (40.30%), hypertension (36.01%), diabetes (15.22%), and hyperlipidemia (11.32%). The top five diseases in terms of prevalence are all cardiometabolic diseases. In this table, two indicators are defined to assess comorbidity patterns. The Overall Comorbidity Rate refers to the proportion of individuals in the total population who have both the primary disease and at least one comorbid condition. This metric provides insight into the prevalence of comorbid cases within the general population. In contrast, the Internal Comorbidity Rate denotes the proportion of individuals with the primary disease who also present with one or more comorbidities. This measure reflects the burden of comorbidity within the subset of patients affected by the primary disease. Hypertension has the highest overall comorbidity rate (26.00%). The internal comorbidity rate for all 21 diseases are over 50%. Among them, depression (93.33%), Alzheimer’s disease (92.86%), and stroke (90.32%) have the highest internal comorbidity rate. Additionally, among diseases with higher prevalence rates, hyperlipidemia (85.03%) and coronary heart disease (87.47%) also have high internal comorbidity rate, indicating that older adult individuals with these two diseases are more likely to have other comorbid conditions.

**Table 2:** Prevalence of each comorbid disease.

Diseases	Patients *N* = 9,811	Patients with other diseases	Prevalence	Overall comorbidity rate	Internal comorbidity rate
Hyperuricemia	3,591	2,120	40.30%	23.79%	59.04%
Hypertension	3,209	2,317	36.01%	26.00%	72.20%
Diabetes	1,356	1,041	15.22%	11.68%	76.77%
Hyperlipidemia	1,009	858	11.32%	9.63%	85.03%
Coronary heart disease	495	433	5.55%	4.86%	87.47%
Arthritis	453	353	5.08%	3.96%	77.92%
Osteoporosis	241	186	2.70%	2.09%	77.18%
Tumors	193	134	2.17%	1.50%	69.43%
Chronic bronchitis	130	97	1.46%	1.09%	74.62%
Stroke	93	84	1.04%	0.94%	90.32%
Migraine	54	44	0.61%	0.49%	81.48%
Myocardial infarction	48	40	0.54%	0.45%	83.33%
Chronic hepatitis	43	28	0.48%	0.31%	65.12%
Asthma	41	34	0.46%	0.38%	82.93%
Nephritis	34	27	0.38%	0.30%	79.41%
Tuberculosis	34	26	0.38%	0.29%	76.47%
Angina pectoris	32	28	0.36%	0.31%	87.50%
Parkinson’s disease	21	12	0.24%	0.13%	57.14%
COPD	18	15	0.20%	0.17%	83.33%
Depression	15	14	0.17%	0.16%	93.33%
Alzheimer’s disease	14	13	0.16%	0.15%	92.86%

Patients with other diseases refers to those who have the listed disease as well as additional comorbidities. Overall comorbidity rate: number of patients with both the disease and any comorbidities/overall population. Internal comorbidity rate: number of patients with both the disease and any comorbidities/patients with the disease.

Table 3 presents the top 10 two-disease and three-disease comorbidity combinations in the overall population, where almost all combinations include cardiometabolic diseases. In addition, Supplementary Figure 4 shows the prevalence rates of all disease combinations in the overall population, with cardiometabolic multimorbidity accounting for a large proportion of the combinations. Specifically, among the top 10 two-disease multimorbidity combinations, hypertension and hyperuricemia appear in five combinations, and their comorbidity have the highest prevalence, reaching 17.26%, far higher than other disease combinations. The prevalence rates of combinations like hypertension and diabetes (8.21%) and hypertension and hyperlipidemia (6.81%) are also high. Additionally, combinations like diabetes and hyperuricemia (6.21%) and hyperlipidemia and hyperuricemia (5.29%) have relatively high prevalence rates. Among the three-disease multimorbidity combinations, aside from the top four highest-ranking metabolic disease combinations, combinations involving coronary heart disease and arthritis along with metabolic diseases are also noteworthy.

**Table 3:** Top 10 multimorbidity combinations.

Comorbidity	Prevalence
Hypertension, Hyperuricemia	17.26%
Hypertension, Diabetes	8.21%
Hypertension, Hyperlipidemia	6.81%
Diabetes, Hyperuricemia	6.21%
Hyperlipidemia, Hyperuricemia	5.29%
Coronary heart disease, Hypertension	3.33%
Diabetes, Hyperlipidemia	3.25%
Coronary heart disease, Hyperuricemia	2.46%
Arthritis, Hypertension	2.08%
Arthritis, Hyperuricemia	2.00%
Hyperuricemia, Hypertension, Diabetes	3.83%
Hyperuricemia, Hypertension, Hyperlipidemia	3.41%
Hypertension, Hyperlipidemia, Diabetes	2.59%
Hyperuricemia, Hypertension, Coronary heart disease	1.63%
Hyperuricemia, Hyperlipidemia, Diabetes	1.41%
Hypertension, Coronary heart disease, Diabetes	1.23%
Hypertension, Hyperlipidemia, Coronary heart disease	0.95%
Hyperuricemia, Hypertension, Arthritis	0.90%
Hyperuricemia, Coronary heart disease, Diabetes	0.77%
Hyperuricemia, Hyperlipidemia, Coronary heart disease	0.63%

Further association rule mining was performed on the overall population, as shown in Supplementary Table 1. The results indicate that hypertension has a strong comorbidity relationship with coronary heart disease (coronary artery disease), hyperlipidemia, and diabetes, with lift values all above 1.50. Hyperuricemia is strongly associated with hypertension and hyperlipidemia, with lift values of 1.19 and 1.16. Additionally, hyperuricemia occurs at a higher rate among patients who have both hypertension and hyperlipidemia (lift value of 1.79). The three-disease combinations of hypertension, diabetes, and hyperuricemia (lift value of 1.71), as well as hypertension, hyperlipidemia, and hyperuricemia (lift value of 1.79), indicate very strong associations among these diseases.

By describing the characteristics and comorbidity combinations in the overall population and performing association rule mining, we found that cardiometabolic multimorbidity accounts for the vast majority of comorbidity combinations, with 1,409 (15.81%) people having cardiometabolic multimorbidity. Hypertension and coronary heart disease, and diabetes and dyslipidemia, have strong comorbidity relationships. Hyperuricemia is shown to accompany the onset of metabolic diseases, with the comorbidity combination of hyperuricemia and hypertension appearing most frequently. In addition, hypertension demonstrates a strong comorbidity capability and may be the most common factor in multimorbidity among the older adult (Figure 2).

### 3.2 Clusters

**Figure 3:**
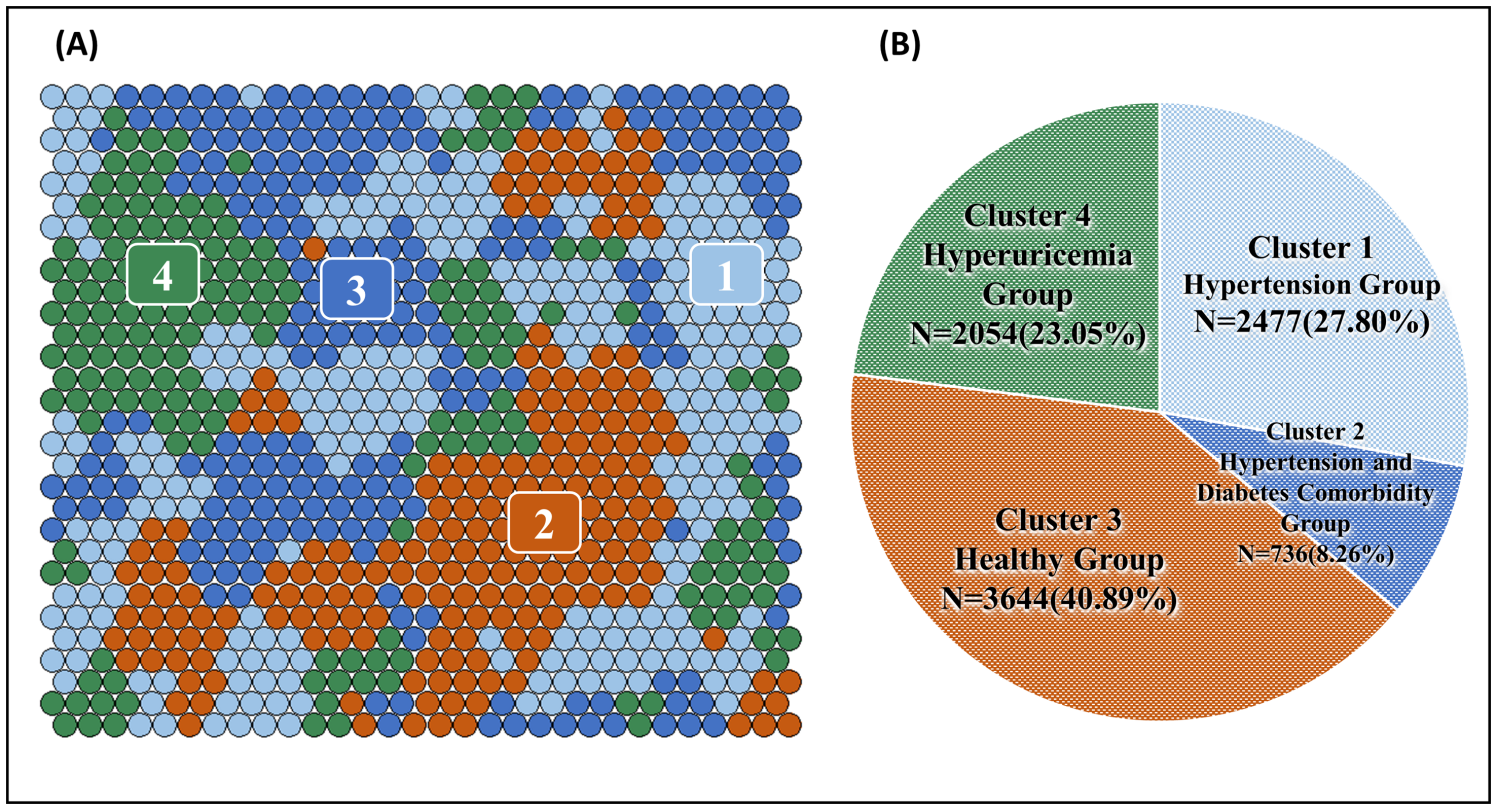
**(A)** Final SOM and **(B)** chart of the number and proportion of the four clusters.

**Figure 4:**
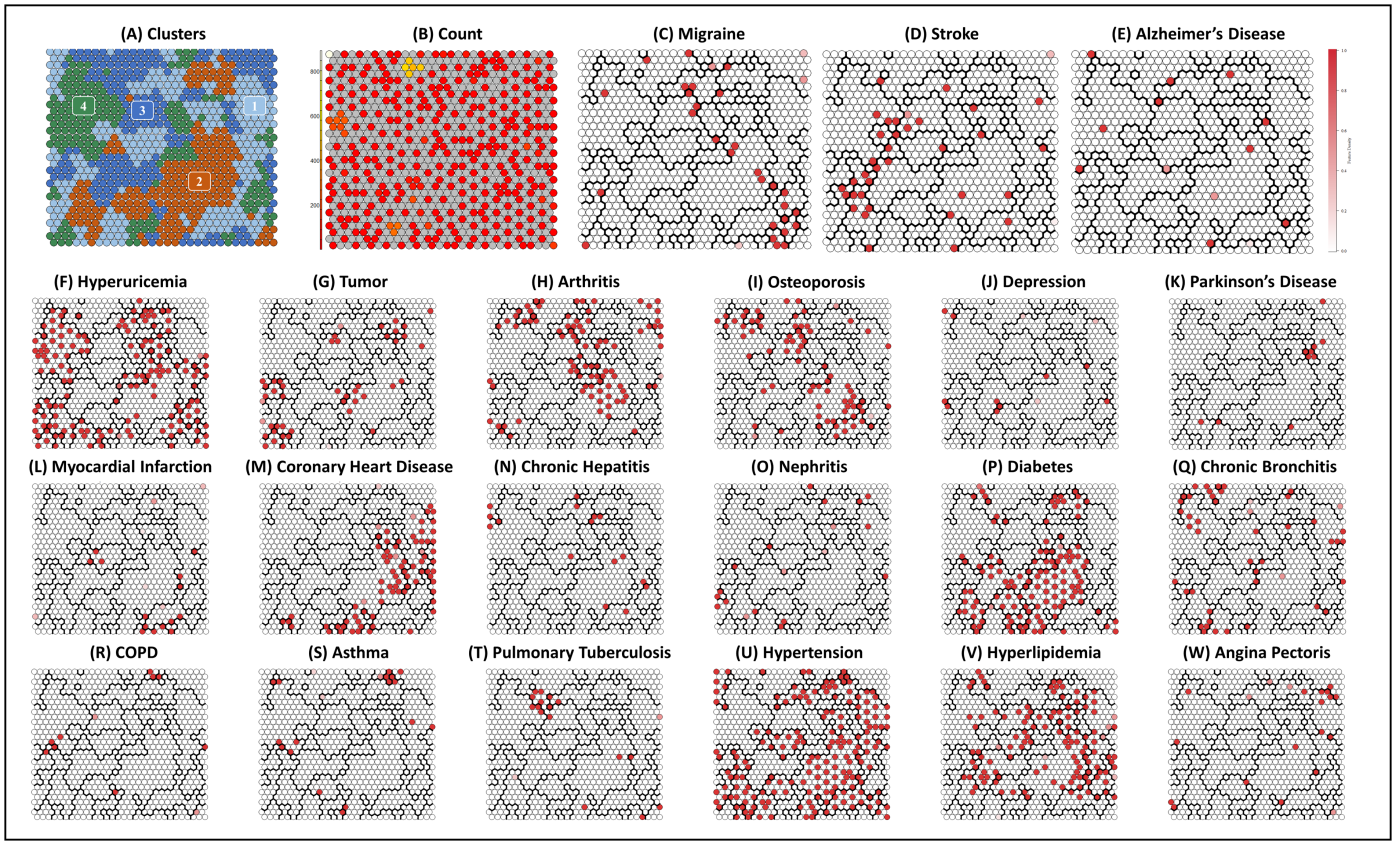
Final SOM and associated cluster boundaries. **(A)** Plot clusters: four clusters by two-level SOM clustering approach. **(B)** Plot count: count of individuals in each node. **(C–W)** Other plots: property heat maps of different diseases categories that show increased density on specific areas of the SOM.

Figures 3A, 4A shows the SOM network and the final output of the four clusters, Figure 3B shows how large each cluster is and each disease is visualized in Figures 4C–W. The count plot in Figure 4B depicts the count of observed individuals in each node; patients are evenly distributed across the map. The characteristics of each cluster are detailed in Table 4. Larger clusters are distributed on the left and top sides. Figures 4C–W respectively show the distribution of different diseases on the map. Due to the inclusion of multiple diseases (mental and physical), the complexity of the high-dimensional disease feature space may be retained in the low-dimensional SOM space, and after sufficient iterations, the data points are relatively scattered.

**Table 4:** Features distribution in four clusters.

	Cluster 1	Cluster 2	Cluster 3	Cluster 4
	Count (prev%)	Count (prev%)	Count (prev%)	Count (prev%)
Sex				
Woman	1,033(41.70%)	315(42.80%)	1,523(41.79%)	938(45.67%)
Man	1,444(58.30%)	421(57.20%)	2,121(58.21%)	1,116(54.33%)
Age				
60–65	710(28.66%)	176(23.91%)	1,367(37.51%)	734(35.74%)
66–70	824(33.27%)	254(34.51%)	1,283(35.21%)	725(35.30%)
71–75	550(22.20%)	169(22.96%)	642(17.62%)	371(18.06%)
76-	393(15.87%)	137(18.61%)	352(9.66%)	224(10.91%)
BMI				
BMI < 18.5	19(0.77%)	6(0.82%)	149(4.09%)	34(1.66%)
18.5 ≤ BMI < 25	1,019(41.14%)	311(42.26%)	2,217(60.84%)	955(46.49%)
25 ≤ BMI < 30	1,216(49.09%)	342(46.47%)	1,172(32.16%)	938(45.67%)
BMI ≥ 30	223(9.00%)	77(10.46%)	106(2.91%)	127(6.18%)
Education				
Primary school and below	437(17.64%)	143(19.43%)	602(16.52%)	356(17.33%)
Junior high school	721(29.11%)	202(27.45%)	962(26.40%)	563(27.41%)
Senior high school	803(32.42%)	235(31.93%)	1,267(34.77%)	722(35.15%)
College or undergraduate	511(20.63%)	155(21.06%)	799(21.93%)	410(19.96%)
Graduate and above	5(0.20%)	1(0.14%)	14(0.38%)	3(0.15%)
Smoke				
No	1,999(80.70%)	578(78.53%)	2,925(80.27%)	1,593(77.56%)
Yes	478(19.30%)	158(21.47%)	719(19.73%)	461(22.44%)
Drink				
No	2,148(86.72%)	640(86.96%)	3,127(85.81%)	1,710(83.25%)
Yes	329(13.28%)	96(13.04%)	517(14.19%)	344(16.75%)

Supplementary Figure 5 includes radar charts for each of the four clusters; radar charts of the same color represent the disease conditions within the same cluster, where the radar chart on the left represents prevalence rates, and the one on the right represents positive rates. Supplementary Figure 6 presents the disease prevalence heatmaps of Clusters 1–4. Table 5 shows the top 10 two-disease and three-disease multimorbidity combinations for the four clusters. Additionally, Supplementary Table 1 contains the association rules for the four clusters.

**Table 5:** Top 10 multimorbidity combinations in four clusters.

Comorbidity	Prevalence
Cluster 1	
Hyperuricemia, Hypertension	48.32%
Hyperlipidemia, Hypertension	15.14%
Hyperlipidemia, Hyperuricemia	8.11%
Hypertension, Coronary heart disease	7.51%
Hypertension, Arthritis	5.93%
Hyperuricemia, Coronary heart disease	3.75%
Hyperuricemia, Arthritis	2.54%
Hypertension, Osteoporosis	2.30%
Hypertension, Tumor	1.98%
Hyperlipidemia, Coronary heart disease	1.86%
Hyperuricemia, Hypertension, Hyperlipidemia	8.11%
Hyperuricemia, Hypertension, Coronary heart disease	3.75%
Hyperuricemia, Hypertension, Arthritis	2.54%
Hypertension, Hyperlipidemia, Coronary heart disease	1.86%
Hypertension, Hyperlipidemia, Arthritis	1.29%
Hyperuricemia, Hyperlipidemia, Coronary heart disease	1.01%
Hyperuricemia, Hypertension, Tumor	0.97%
Hyperuricemia, Hypertension, Osteoporosis	0.93%
Hyperuricemia, Hypertension, Stroke	0.69%
Hypertension, Osteoporosis, Arthritis	0.69%
Cluster 2	
Diabetes, Hypertension	99.32%
Hypertension, Hyperuricemia	46.33%
Diabetes, Hyperuricemia	46.20%
Diabetes, Hyperlipidemia	31.93%
Hyperlipidemia, Hypertension	31.52%
Diabetes, Coronary heart disease	15.49%
Coronary heart disease, Hypertension	15.08%
Hyperlipidemia, Hyperuricemia	13.99%
Coronary heart disease, Hyperuricemia	7.07%
Coronary heart disease, Hyperlipidemia	5.84%
Hyperuricemia, Hypertension, Diabetes	46.20%
Hypertension, Hyperlipidemia, Diabetes	31.39%
Hypertension, Coronary heart disease, Diabetes	14.95%
Hyperuricemia, Hypertension, Hyperlipidemia	13.99%
Hyperuricemia, Hyperlipidemia, Diabetes	13.86%
Hyperuricemia, Hypertension, Coronary heart disease	7.07%
Hyperuricemia, Coronary heart disease, Diabetes	6.93%
Hyperlipidemia, Coronary heart disease, Diabetes	5.71%
Hypertension, Hyperlipidemia, Coronary heart disease	5.30%
Hypertension, Diabetes, Arthritis	5.16%
Cluster 3	
Diabetes, Hyperlipidemia	0.85%
Arthritis, Osteoporosis	0.60%
Coronary heart disease, Hyperlipidemia	0.52%
Diabetes, Coronary heart disease	0.49%
Osteoporosis, Hyperlipidemia	0.47%
Arthritis, Diabetes	0.47%
Arthritis, Hyperlipidemia	0.44%
Arthritis, Coronary heart disease	0.27%
Tumor, Diabetes	0.27%
Arthritis, Chronic bronchitis	0.25%
Hyperlipidemia, Osteoporosis, Arthritis	0.14%
Chronic bronchitis, Hyperlipidemia, Diabetes	0.11%
Diabetes, Osteoporosis, Arthritis	0.11%
Hyperlipidemia, Migraine, Osteoporosis	0.08%
Chronic bronchitis, Hyperlipidemia, Osteoporosis	0.05%
Chronic bronchitis, Hyperlipidemia, Arthritis	0.05%
Chronic bronchitis, Diabetes, Arthritis	0.05%
Hyperlipidemia, Coronary heart disease, Osteoporosis	0.05%
Hyperlipidemia, Diabetes, Stroke	0.05%
Hyperlipidemia, Diabetes, Osteoporosis	0.05%
Cluster 4	
Diabetes, Hyperuricemia	10.32%
Hyperlipidemia, Hyperuricemia	8.13%
Arthritis, Hyperuricemia	4.77%
Coronary heart disease, Hyperuricemia	3.60%
Osteoporosis, Hyperuricemia	2.24%
Tumor, Hyperuricemia	1.56%
Chronic bronchitis, Hyperuricemia	1.41%
Diabetes, Hyperlipidemia	1.17%
Diabetes, Coronary heart disease	0.88%
Coronary heart disease, Hyperlipidemia	0.78%
Hyperuricemia, Hyperlipidemia, Diabetes	1.17%
Hyperuricemia, Coronary heart disease, Diabetes	0.88%
Hyperuricemia, Hyperlipidemia, Coronary heart disease	0.78%
Hyperuricemia, Hyperlipidemia, Arthritis	0.73%
Hyperuricemia, Osteoporosis, Arthritis	0.73%
Hyperuricemia, Diabetes arthritis	0.49%
Hyperuricemia, Hyperlipidemia, Osteoporosis	0.44%
Hyperuricemia, Chronic bronchitis, Diabetes	0.24%
Hyperuricemia, Diabetes, Osteoporosis	0.24%
Hyperlipidemia, Osteoporosis, Arthritis	0.24%

Overall, unsupervised learning provides us with a new perspective to classify multimorbidity patterns at the population level. Older adult residents in Shenzhen can be divided into the Hypertension Group, Hypertension and Diabetes Comorbidity Group, Healthy Group, and Hyperuricemia Group. The description of the characteristics and comorbidity combinations of the four clusters, along with association rule mining, revealed a more specific distribution of comorbidity combinations, further confirming the prevalent comorbidity between hyperuricemia and cardiometabolic diseases (such as diabetes, hypertension, hyperlipidemia, and coronary heart disease).

Furthermore, this study examined inter-cluster differences in three fundamental demographic and health-related variables: gender, age, and BMI. Chi-square tests were used to assess differences in gender distribution across clusters, followed by pairwise Chi-square comparisons. For the continuous variables—age and BMI—one-way analysis of variance (ANOVA) was first performed to detect overall differences, and *post hoc* comparisons between clusters were conducted using the Tukey HSD test. The following section presents a descriptive summary of each cluster. Below is a descriptive summary of each cluster:

Cluster 1 (Hypertension Group): this group had a gender ratio similar to the overall population. The average age was 68.51 ± 5.68 years, which was significantly higher than that of Cluster 3 (*p* < 0.001), but significantly lower than Cluster 2 (*p* < 0.05); no significant difference was found compared to Cluster 4. The group exhibited a relatively high BMI, with significantly higher values than Cluster 3 (*p* < 0.001), though similar to Cluster 2 (*p* = 0.966). The prevalence of overweight and obesity was relatively high. The age distribution was balanced. Educational attainment was higher compared to other clusters, and the proportions of smokers and drinkers were relatively low. All individuals in this group had hypertension; nearly half (48.32%) also had hyperuricemia, and a portion were diagnosed with hyperlipidemia.

Cluster 2 (Hypertension and Diabetes Comorbidity Group): this group also had a gender ratio similar to the overall population. The average age was 69.11 ± 5.63 years, the highest among all clusters, and was significantly greater than Clusters 1, 3, and 4 (*p* < 0.001). The BMI was relatively high, with most individuals falling into the overweight or normal categories. BMI in this group was significantly higher than in Cluster 3 (*p* < 0.001), similar to Cluster 1 (*p* = 0.966), and significantly lower than Cluster 4 (*p* < 0.05). Educational level was relatively lower, and the proportions of smokers and drinkers were slightly higher than in Cluster 1. Nearly all individuals (99.32%) had comorbidity of hypertension and diabetes; 46.20% had co-occurrence of hyperuricemia, hypertension, and diabetes. More than one-quarter had hyperlipidemia.

Cluster 3 (Healthy Group): this was the largest cluster, with a gender ratio similar to the overall population. The average age was 67.07 ± 5.11 years, making it the youngest group. This value was significantly lower than in all other clusters (*p* < 0.001). BMI was predominantly within the normal range and was significantly lower than in Clusters 1, 2, and 4 (all *p* < 0.001), representing the lowest among the clusters. This group had a relatively high level of educational attainment. The proportions of smokers and drinkers were moderate. Compared to other clusters, this group exhibited the lowest prevalence of chronic diseases and the lowest rates of multimorbidity.

Cluster 4 (Hyperuricemia Group): this group had a slightly higher proportion of females than the overall population. According to chi-square tests, the gender distribution was significantly different from that of Clusters 1 and 3 (*p* < 0.01). The average age was 67.41 ± 5.34 years, significantly higher than Cluster 3 (*p* < 0.001), but lower than Clusters 1 (*p* < 0.05) and 2 (*p* < 0.001). BMI was mostly within the normal and overweight categories and was significantly higher than in all other clusters (*p* < 0.05). Educational attainment was relatively lower. The proportions of smokers and drinkers were lower than those of non-smokers and non-drinkers. All individuals in this group had hyperuricemia, and the most common comorbidities involved hyperuricemia combined with other chronic diseases.

## 4 Discussion

This study conducted a cross-sectional analysis based on the baseline data of the Shenzhen older adult cohort. Utilizing information on the prevalence and multimorbidity of 21 diseases among the older adult in Shenzhen, we employed the Kohonen method combined with weighted k-means clustering to visualize the distribution of diseases within the population. Based on this visualization, we divided the overall population into four representative disease clusters. Furthermore, we described the common comorbidity combinations in both the overall population and each cluster and performed association rule mining. The results revealed a high prevalence of cardiometabolic diseases and their comorbidities, as well as common combinations of these diseases. Hyperuricemia was found to occur alongside metabolic diseases. Hypertension exhibited the strongest comorbidity tendency and may be a common cause of multimorbidity.

Compared with previous studies on multimorbidity in Shenzhen, this paper places a greater emphasis on exploring multimorbidity patterns. We employed a two-stage clustering method using Self-Organizing Maps to investigate the distribution of different diseases within the population. Innovatively, this study used the activation frequency of each node in the SOM network as weights in the weighted k-means algorithm, aiming to better partition the population. We obtained three representative clusters: the first cluster consists entirely of hypertensive patients; the second cluster predominantly comprises patients with both hypertension and diabetes; and the third cluster is the hyperuricemia group. From these disease clusters, we can observe the multimorbidity patterns among the older adult in Shenzhen. After partitioning the comorbidity clusters at the population level, we further described the combinations in the overall population and within each cluster, and conducted association rule mining to further analyze the multimorbidity patterns.

This study found a high prevalence of cardiometabolic multimorbidity among the older adult in Shenzhen, reaching 15.83%, and demonstrated a more detailed distribution of comorbidity combinations. In a study conducted in Chongqing, China, 11.2% of middle-aged and older adult individuals had CMM, with the most common cardiometabolic disease being hypertension (16.5%), followed by dyslipidemia (15.1%) and diabetes (6.4%) (16). In another study using CLHLS data, 7.0% of participants had cardiometabolic multimorbidity (39). Similar patterns of cardiometabolic multimorbidity have also been observed in some studies analyzing multimorbidity patterns. In a study on multimorbidity among the older adult in China, the dyslipidemia/diabetes/hypertension/coronary heart disease/kidney disease pattern was the most common, with a prevalence of 22.4% (40). Another study classified 18.60% of patients under vascular system diseases such as hypertension, dyslipidemia, diabetes, heart disease, and stroke (41). Research indicates that hypertensive patients with two or more cardiometabolic diseases have significantly increased risks of all-cause mortality and cardiovascular mortality. Cohort studies have shown that in China, the prevalence of cardiometabolic multimorbidity has been increasing annually, more than doubling within 5 years, suggesting that cardiometabolic diseases are rapidly developing (42). Shenzhen should implement further management for this high-risk patient population.

In this study, we found that the prevalence of hypertension is very high, having the highest comorbidity rate, and it is the most common factor in multimorbidity among the older adult in Shenzhen. A previous study in Shenzhen also found that hypertension had the top four comorbidity prevalence rates with chronic pain, diabetes, hyperlipidemia, and bone diseases. Hypertension often coexists with multiple comorbidities. A review of the prevalence and patterns of multimorbidity in China showed that hypertension paired with hearing impairment, dyslipidemia, diabetes, eye diseases, and obesity constituted the five major multimorbidity patterns; other disease pairs also included hypertension, suggesting it may be the most common component in multimorbidity (14). In another study exploring cardiometabolic comorbidities among hypertensive patients in China, it was found that three-quarters of hypertensive patients had cardiometabolic comorbidities (43). In the UK Biobank database, 70, 64, and 57% of patients with chronic kidney disease (CKD), diabetes mellitus (DM), and stroke were also diagnosed with hypertension (44). A clinical study indicated that maintaining blood pressure control might be an effective method to slow the progression of comorbidities and could potentially reduce the population burden of multimorbidity (45).

Hyperuricemia was included as a disease in this study to explore its multimorbidity patterns with various other chronic conditions. Hyperuricemia is a metabolic disorder associated with gout, cardiovascular diseases, kidney diseases, and other ailments, and its global prevalence has been rising in recent years (46). Gout, as a metabolic disorder characterized by hyperuricemia and marked by prominent symptoms such as painful inflammatory arthritis, has been the subject of extensive research due to its high prevalence and substantial burden of comorbidities (47–49). In contrast, although hyperuricemia has been widely studied for its associations with various cardiovascular and metabolic diseases (47), relatively few studies have investigated the comorbidity patterns of hyperuricemia with other diseases at the population level.

In this study, we found that hyperuricemia among the older adult in Shenzhen often co-occurs with metabolic diseases. Another study from Guangdong Province found that dyslipidemia and hyperuricemia, and hyperuricemia and hypertension ranked first and third, respectively, among binary multimorbidity combinations. Among ternary multimorbidity combinations, dyslipidemia, hyperuricemia, and hypertension were the most common disease combination among middle-aged and older adult populations (50). Elevated serum uric acid levels are associated with the development of metabolic diseases (51). Compared to subjects with normal uric acid levels, patients with hyperuricemia have a 2.10-fold increased risk of metabolic syndrome (MetS) (PR = 2.10, 95% CI: 1.68–2.63). Among these, the association between high triglycerides and hyperuricemia is the strongest (PR = 2.32, 95% CI: 1.84–2.91) (52). A cohort study showed that over a 7-year follow-up, participants who maintained or progressed to hyperuricemia had a 1.86 times higher chance (95% CI: 1.29, 2.68) of progressing to cardiometabolic multimorbidity compared to those who maintained or reduced to non-hyperuricemia status (53). Therefore, uric acid control should be included in the formulation of chronic disease prevention and control policies in Shenzhen.

In recent years, the Chinese government has released a series of policy documents emphasizing the implementation of integrated chronic disease prevention and control strategies, as well as the improvement of health management efficiency. In 2020, China launched the “Three Highs” co-management initiative, which focuses on hypertension, hyperglycemia, and hyperlipidemia as key entry points for exploring new models of chronic disease prevention and control. While several international guidelines on comorbidity management have been established, there is currently a lack of corresponding guidelines specifically tailored to comorbidities in China (54). Managing comorbid conditions presents numerous challenges, particularly the transition from traditional single-disease treatment approaches to care models that address multiple coexisting conditions. Comorbidity management requires patient-centered and family-centered care, along with a consideration of individual patients’ care goals—indicating that there is no one-size-fits-all approach to the management of multiple chronic conditions. This study identifies representative subgroups among older adult individuals with comorbidities in Shenzhen, shedding light on the heterogeneity of chronic disease populations in the community (2). These findings provide both a theoretical foundation and practical direction for precision health management. Further attention to the healthcare needs and care practices of these representative subgroups will help facilitate the development of more effective strategies for comorbidity management and nursing care.

Based on the identified four clusters, targeted stratified management and intervention strategies can be envisioned:

- Cluster 1 (Hypertension Group): individuals in this group may benefit from enhanced blood pressure monitoring, improved medication adherence, and weight reduction interventions, aiming to prevent progression toward more complex metabolic comorbidities.
- Cluster 2 (Hypertension and Diabetes Comorbidity Group): this group should be considered a key target for chronic disease management. A multidisciplinary team (MDT) approach is recommended, focusing on integrated control of blood pressure, blood glucose, body weight, and lipid levels. Concurrently, behavioral interventions such as smoking cessation, alcohol restriction, dietary modification, and physical activity prescriptions should be reinforced.
- Cluster 3 (Healthy Group): as a relatively healthy population, this group is suited for preventive management strategies. Regular health check-ups and digital health interventions (e.g., apps and tracking systems) are encouraged, with an emphasis on early identification and management of sub-health conditions.
- Cluster 4 (Hyperuricemia Group): greater attention should be paid to the identification and management of hyperuricemia, particularly among women. Nutritional guidance and lifestyle modifications are essential to prevent the development of other chronic conditions such as hypertension and diabetes.

In addition, cluster labels derived from unsupervised learning may serve as important markers for future personalized healthcare. These labels could be integrated into community-based electronic health records or chronic disease follow-up systems, helping healthcare providers quickly assess individual risk levels. Moreover, the clustering results can inform resource allocation strategies. For instance, public health resources might be prioritized for Cluster 2 and Cluster 4 populations to enhance the effectiveness and cost-efficiency of interventions.

This study has limitations. As a cross-sectional study, it cannot assess causal relationships. Data compilation for the Shenzhen older adult cohort is still ongoing, and we look forward to future cohort studies better illustrating the progression of multimorbidity among the older adult in Shenzhen. Nevertheless, the evidence from this study can still provide useful information for the formulation of health-related policies and the allocation of social health services. The findings may also serve as a reference for similar cities in other countries.

## 5 Conclusion

Multimorbidity is prevalent among the older adult population in Shenzhen. The use of the SOM method and weighted k-means clustering effectively characterizes the health status of individuals within this group. The results indicate that hyperuricemia, along with hypertension, diabetes, hyperlipidemia, coronary heart disease, and other cardiometabolic diseases, imposes a heavy burden. Hypertension may be a common cause of these comorbidities. Shenzhen should strengthen screening of high-risk populations and enhance comprehensive management of multimorbidity. Public health interventions should be implemented to alleviate the burden of multimorbidity.

## Data Availability

The original contributions presented in the study are included in the article/Supplementary material, further inquiries can be directed to the corresponding authors.

## Supplementary material

SUPPLEMENTARY FIGURE 1 Elbow plot of the total within-cluster sum of squares (WSS) for varying numbers of clusters.

SUPPLEMENTARY FIGURE 2 Prevalence of different features across diseases. Each cell represents the prevalence of the feature in the disease patients of the corresponding column, calculated as: (Number of patients with the feature in the disease / Total number of patients with the disease in the column) × 100%. Values below 0.1% are hidden.

SUPPLEMENTARY FIGURE 3 Prevalence of different features across diseases. Same as Figure 2.

SUPPLEMENTARY FIGURE 4 Multimorbidity prevalence between pairs of diseases. Each cell represents the comorbidity prevalence between the row and column diseases, calculated as: (Number of comorbid patients / Total study population) × 100%. Values below 0.1% are hidden.

SUPPLEMENTARY FIGURE 5 Radar plots for four clusters with diseases. Left radar charts: Disease prevalence within each cluster, calculated as: (Number of patients with the disease in the cluster / Total cluster population) × 100%. Right radar charts: Positive rate of the disease in the cluster relative to the overall population, calculated as: (Number of patients with the disease in the cluster / Total patients with the disease in the overall population) × 100%.

SUPPLEMENTARY FIGURE 6 Multimorbidity prevalence between diseases within four clusters. Each cell represents comorbidity prevalence within the cluster, calculated as: (Number of comorbid patients in the cluster / Total cluster population) × 100%. Values below 0.1% are hidden.

SUPPLEMENTARY TABLE 1 Overall association rule mining results.

SUPPLEMENTARY TABLE 2 Association rule mining results stratified by four clusters.
