# Supplementary figures and images for: An unsupervised cluster analysis of multimorbidity patterns in older adults in Shenzhen, China
Source: Front Public Health. 2025 Jun 6;13:1557721. doi: 10.3389/fpubh.2025.1557721 (PMC12180302; doi:10.3389/fpubh.2025.1557721)

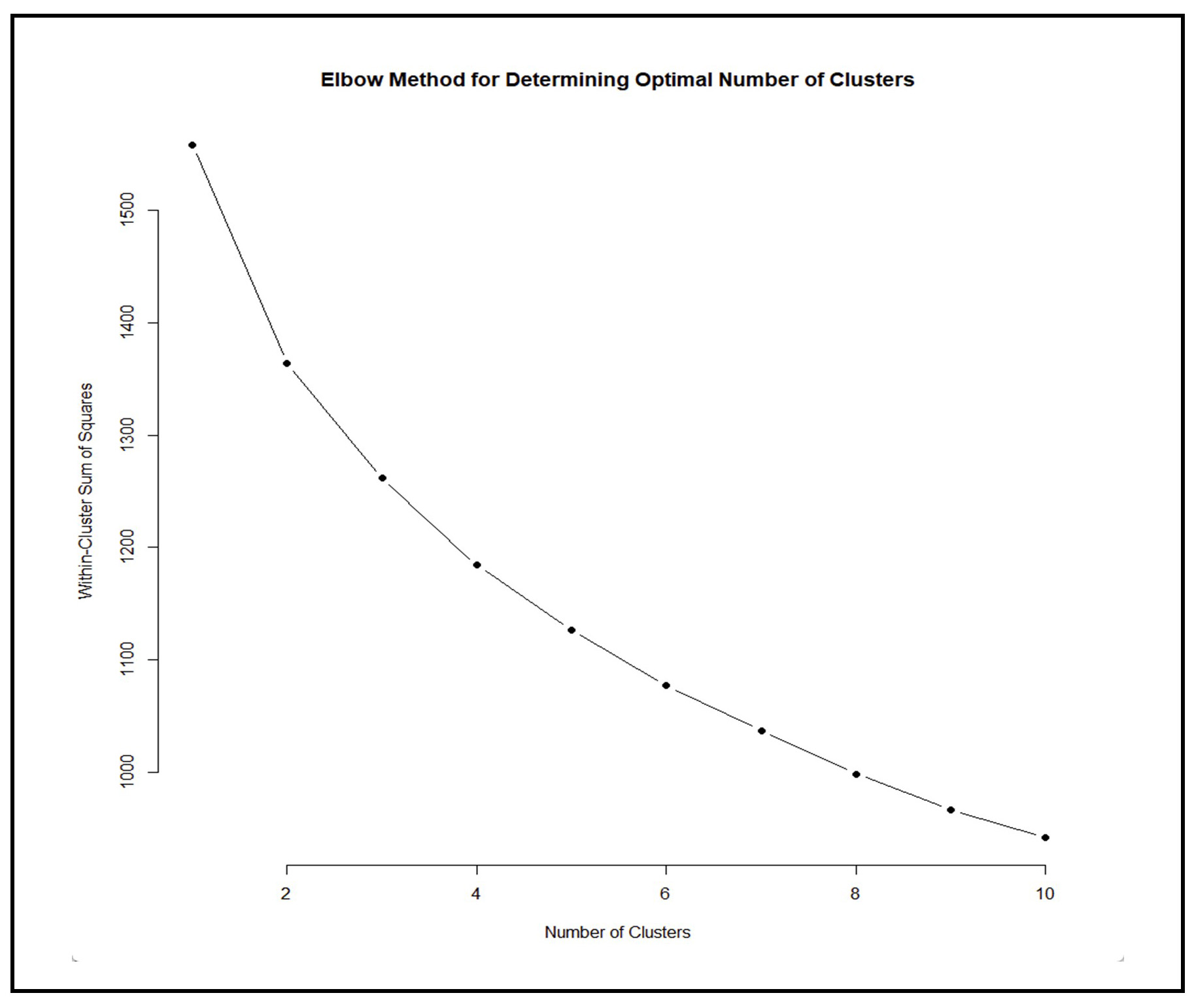

Supplement: SUPPLEMENTARY FIGURE 1 — Elbow plot of the total within-cluster sum of squares (WSS) for varying numbers of clusters. [file Image_1.JPEG]

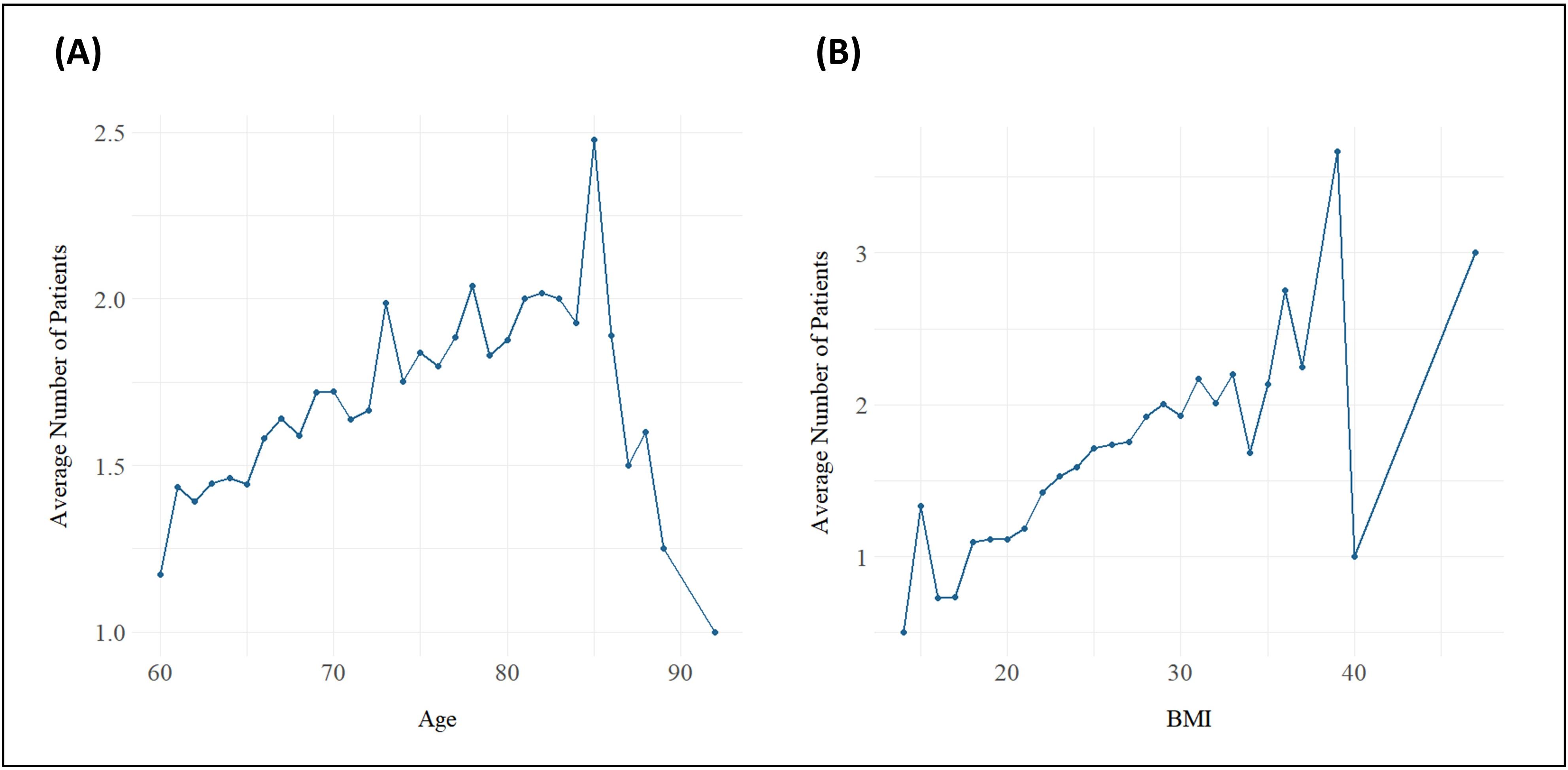

Supplement: SUPPLEMENTARY FIGURE 2 — Prevalence of different features across diseases. Each cell represents the prevalence of the feature in the disease patients of the corresponding column, calculated as: (Number of patients with the feature in the disease / Total number of patients with the disease in the column) × 100%. Values below 0.1% are hidden. [file Image_2.JPEG]

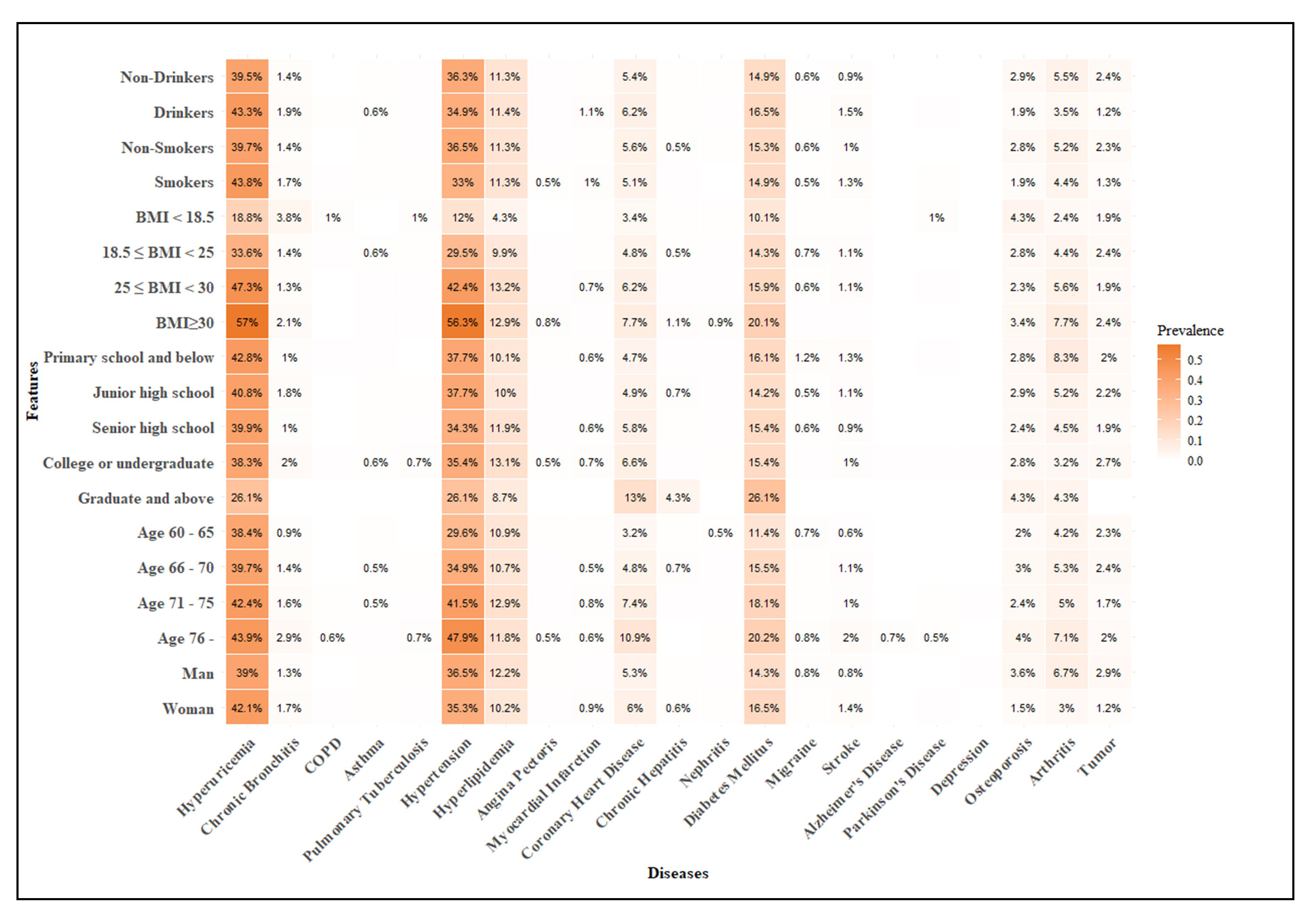

Supplement: SUPPLEMENTARY FIGURE 3 — Prevalence of different features across diseases. Same as Figure 2. [file Image_3.JPEG]

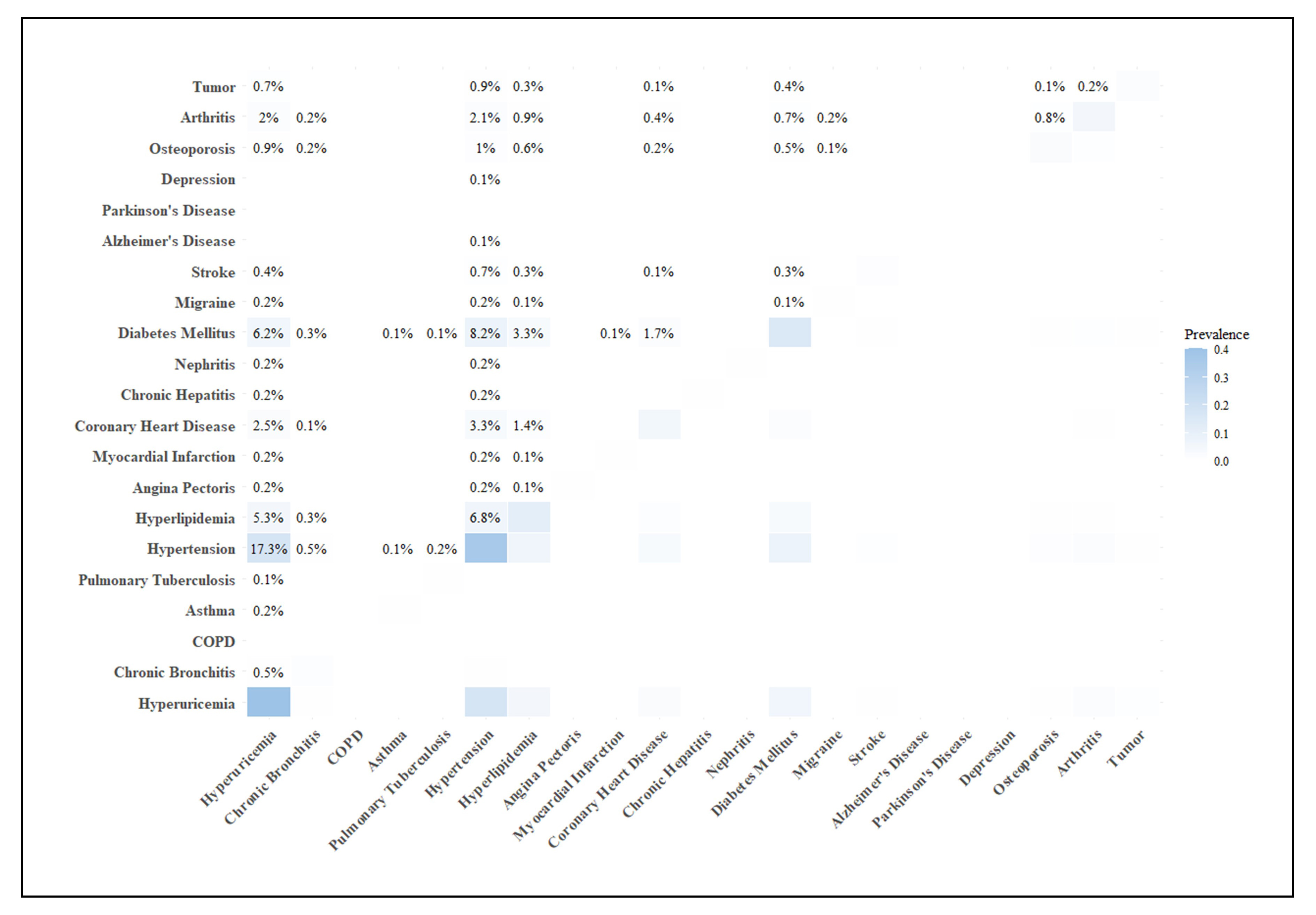

Supplement: SUPPLEMENTARY FIGURE 4 — Multimorbidity prevalence between pairs of diseases. Each cell represents the comorbidity prevalence between the row and column diseases, calculated as: (Number of comorbid patients / Total study population) × 100%. Values below 0.1% are hidden. [file Image_4.JPEG]

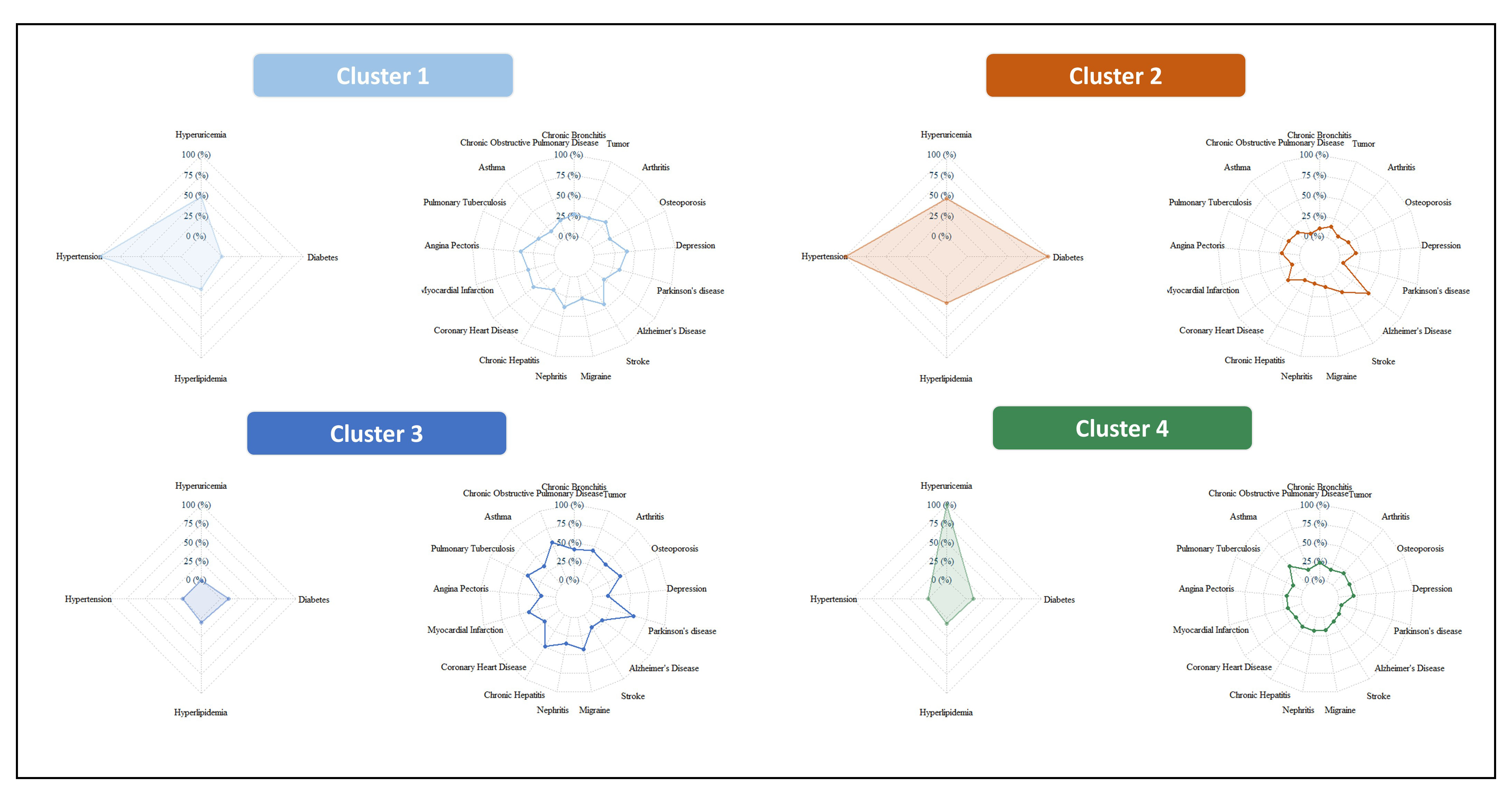

Supplement: SUPPLEMENTARY FIGURE 5 — Radar plots for four clusters with diseases. Left radar charts: Disease prevalence within each cluster, calculated as: (Number of patients with the disease in the cluster / Total cluster population) × 100%. Right radar charts: Positive rate of the disease in the cluster relative to the overall population, calculated as: (Number of patients with the disease in the cluster / Total patients with the disease in the overall population) × 100%. [file Image_5.JPEG]

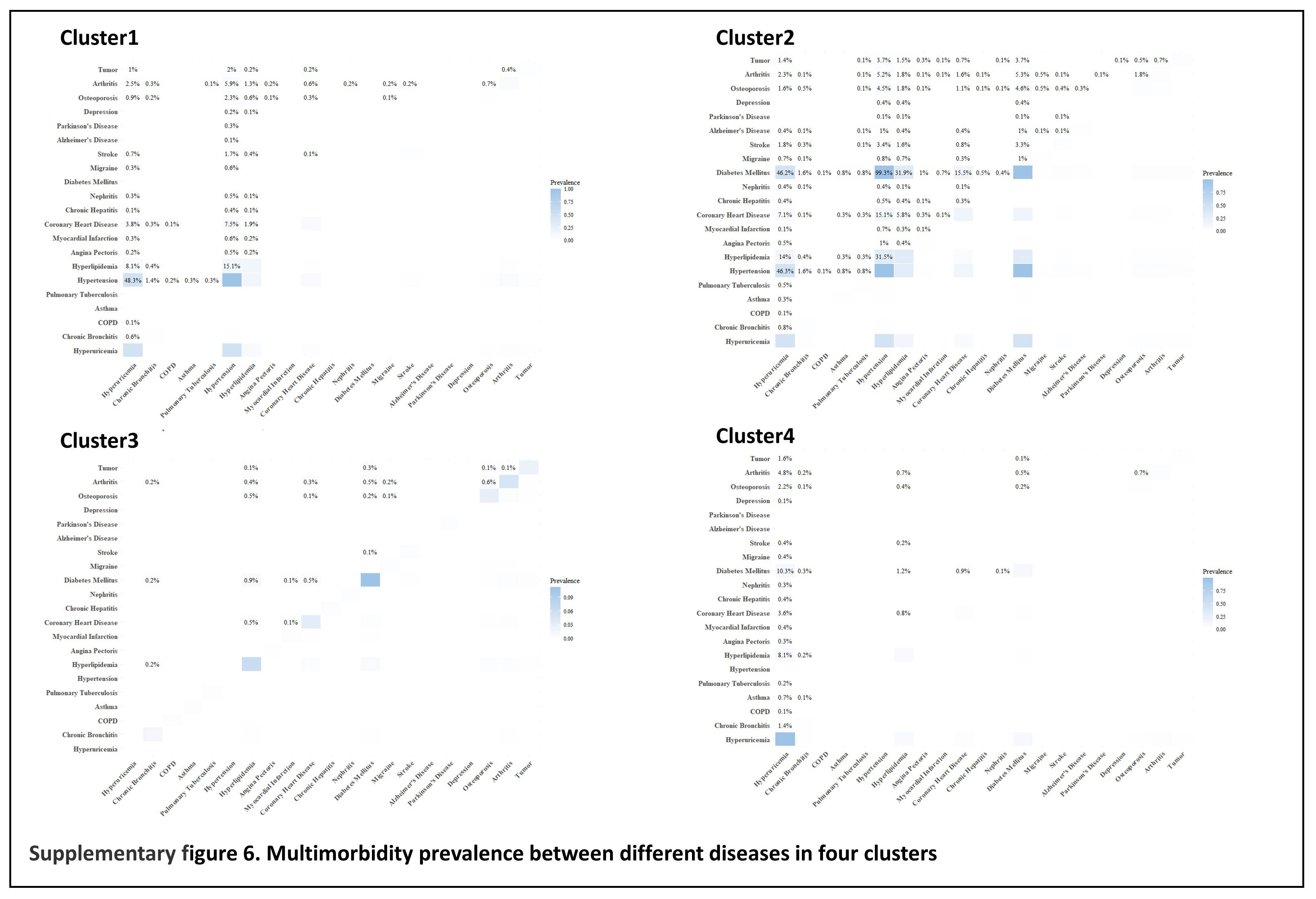

Supplement: SUPPLEMENTARY FIGURE 6 — Multimorbidity prevalence between diseases within four clusters. Each cell represents comorbidity prevalence within the cluster, calculated as: (Number of comorbid patients in the cluster / Total cluster population) × 100%. Values below 0.1% are hidden. [file Image_6.JPEG]
